# Chemically Based Mathematical Model for Development of Cerebral Cortical Folding Patterns

**DOI:** 10.1371/journal.pcbi.1000524

**Published:** 2009-09-25

**Authors:** Deborah A. Striegel, Monica K. Hurdal

**Affiliations:** Department of Mathematics, Florida State University, Tallahassee, Florida, United States of America; University College London, United Kingdom

## Abstract

The mechanism for cortical folding pattern formation is not fully understood. Current models represent scenarios that describe pattern formation through local interactions, and one recent model is the intermediate progenitor model. The intermediate progenitor (IP) model describes a local chemically driven scenario, where an increase in intermediate progenitor cells in the subventricular zone correlates to gyral formation. Here we present a mathematical model that uses features of the IP model and further captures *global* characteristics of cortical pattern formation. A prolate spheroidal surface is used to approximate the ventricular zone. Prolate spheroidal harmonics are applied to a Turing reaction-diffusion system, providing a chemically based framework for cortical folding. Our model reveals a direct correlation between pattern formation and the size and shape of the lateral ventricle. Additionally, placement and directionality of sulci and the relationship between domain scaling and cortical pattern elaboration are explained. The significance of this model is that it elucidates the consistency of cortical patterns among individuals within a species and addresses inter-species variability based on global characteristics and provides a critical piece to the puzzle of cortical pattern formation.

## Author Summary

The size and shape of the cerebral cortex varies across species. The cortical folding pattern also varies from a smooth surface where no pattern is visible, as observed in the common treeshrew (*Tupaia glis*) and Eastern mole (*Scalopus aquaticus*), to an intricate labyrinthine pattern, as observed in humans. One current model, the intermediate progenitor model, describes the creation of a fold through local interactions in the ventricular zone which surrounds the lateral ventricle. Here we extend the local scenario described in the intermediate progenitor model to include global characteristics that differ between species. We approximate the lateral ventricle with a prolate spheroid and examine how patterns on a spheroidal surface change based on size and eccentricity. Our model reveals a direct correlation between pattern formation and lateral ventricular size and shape. This model's significance is that it elucidates the consistency of cortical patterns among individuals within a species and addresses inter-species variability based on global characteristics, such as size and shape of the lateral ventricle, and provides a critical piece to the puzzle of cortical pattern formation.

## Introduction

Cerebral cortical patterns have fascinated scientists for centuries with their beauty and complexity. Numerous groups relate malformations in sulcal patterns to different diseases in humans, such as autism [1] and attention deficit/hyperactivity disorder (ADHD) [2]. Though many advances have occurred in cortical development and sulcogenesis, the understanding of how sulci form and what factors determine the placement of sulci is still limited. The cerebral cortex across species displays a variety of shapes and sizes and also wide array of sulcal patterning. Studying the evolutionary development of sulcal patterns might provide clues about the cortical development taking place in humans.

A major advance in determining how these sulcal patterns form was the introduction of the axonal tension hypothesis [3]. This hypothesis describes a mechanically-based scenario where axonal tension, created by developing corticocortical connections in strongly interconnected regions, pulls together gyral walls and creates a folding pattern. This hypothesis furthered the concept that variability between folding patterns among individuals is genetically driven, not just the consequence of random mechanical buckling from a confined cortex. Other mechanochemical models have also been proposed to explain morphogenesis in the central nervous system [4].

Recently, it has been suggested that a cortical pattern can arise based on regional patterns of intermediate progenitor (IP) cells in the subventricular zone (SVZ) [5]. The intermediate progenitor model, which builds upon the intermediate progenitor cell hypothesis [6], states that during the development of the cortex certain radial glial cells in the ventricular zone (VZ) are activated to create IP cells that travel to the SVZ. These IP cells amplify the amount of neurons created in a given radial column. Furthermore, a subset of IP cells creates a local amplification of neurons in upper cortical layers surrounded by areas of non-amplification, resulting in a wedge shape in the cortex. This wedge shape is representative of a gyrus. This new hypothesis is still being debated [7],[8] and, if correct, could be a scenario for chemically-based pattern formation in the cortex.

Here, a relatively simple and, we believe, elegant chemically-driven mathematical model is proposed to explain how IP cell subsets are distributed spatially and temporally in the developing cortex. Our model, which we call the Global Intermediate Progenitor (GIP) model, uses a Turing reaction-diffusion system [9] containing an activator and inhibitor on a prolate spheroidal surface to determine regional areas of activation of the production of IP cells. The GIP model allows determination of the placement of the initial sulci underlying observed complex cortical patterns. It also demonstrates that the initial folds of the arising sulcal pattern are governed by the global shape of the lateral ventricle. The dependency on the global shape provides a critical piece to the puzzle of cortical development.

## Model

In the Intermediate Progenitor Model, sulci occur based on the distribution of IP cells during cortical development. Mutations of *Pax6*, *Ngn2*, and *Id4* have been shown to increase the production of IP cells in mice [10]. Thus it is not unreasonable to assume that there is an activator and inhibitor located in the VZ controlling the production of IP cells.

**Figure 1:**
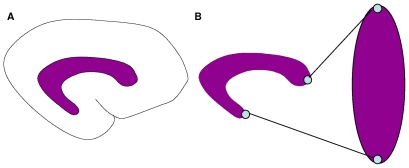
Approximation of lateral ventricle with prolate spheroid. **A.** Position of lateral ventricle with respect to cerebral cortex. **B.** The shape of the lateral ventricle can be approximated with a prolate spheroid. The poles of the anterior and inferior horns map to the north and south poles of the prolate spheroid, respectively.

We assume an activator and inhibitor are travelling throughout the VZ, which is located in the lateral wall of the lateral ventricle. The lateral ventricle is a *c*-shaped cavity located in both hemispheres of the cerebral cortex [11] as shown in Figure 1. The proposed GIP model approximates the shape of the lateral ventricle with a prolate spheroid and the VZ with a prolate spheroidal surface. This allows the capture of key domain shape characteristics (the eccentricity of the lateral ventricle) with the focal distance (*f*) of a prolate spheroid.

Also, there are multiple discrete time intervals where the distribution of the reactants determines the creation of IP cells. These time intervals represent the temporal windows [5] for the production of neurons in the upper cortical layers. This assumption allows for the layering of multiple patterns to form based on distributions of an activator and inhibitor.

### Reaction-Diffusion System

The patterns created by Turing reaction-diffusion systems have been used to describe pattern formation in numerous biological systems [12]. Though biological Turing patterns have not been proven as rigorously as chemical Turing patterns [13], recent results [14] give supporting evidence of Turing patterns formation in a biological setting. A Turing system is a reaction-diffusion system, given by
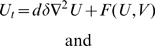
(1a)

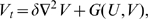
(1b)containing an activator (*U*) and an inhibitor (*V*) that are diffusing throughout their domain and interacting with each other as described by the reaction kinetics (*F* and *G*). The reaction kinetics chosen for the GIP model are from the Barrio-Varea-Maini (BVM) [15] system given by
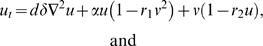
(2a)

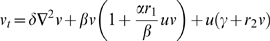
(2b)where (*u*, *v*) = (*U*−*U*_0_, *V*−*V*_0_) and (*U*_0_, *V*_0_) is the steady state. Not much is known about the possible interactions between *u* and *v* that regulate the production of IP cells. Hence the BVM system is ideal because the kinetic equations do not assume any prior knowledge of how the reactants (activator and inhibitor) interact and instead takes a phenomenological approach. These kinetics (Equations 2a,b) also provide control over the amount of linear (α and γ for *u*; and β for *v*), quadratic (*r*_2_), and cubic (*r*_1_) interactions. The diffusivity ratio and domain scaling are given by *d* and δ, respectively. Key aspects of the system that determine what pattern will arise include the ratio of the diffusivities of the activator and inhibitor, domain scale and shape, and quadratic versus cubic terms in the kinetic reactions [12],[16].

In order to analyze the nonlinear BVM system, the kinetics are approximated linearly by expanding them in a Taylor series around the steady state and neglecting higher order terms resulting in
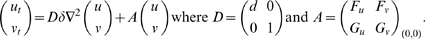
(3)Solutions of Equation 3 are of the form (*u*, *v*) = **T**(t)**X**(**x**). The temporal solution is T(t) = e^λ(k^2^^ ^)t^, where λ(k^2^) is the temporal eigenvalue. The spatial solution solves the Helmholtz Equation (**∇** ^2^ **X**+*k* ^2^ **X** = 0) in the given domain where *k* ^2^ is the spatial eigenvalue.

Turing patterns have been studied in depth in 1D [12], 2D [17], and spherical domains [18]. In all these domains, the solution to the domain's associated eigenvalue problem can predict which pattern will form. For a spherical domain, the eigenvalue solution yields *k* ^2^ = *n*(*n*+1)/*r* ^2^, where *n* is the spherical harmonic index and *r* is the radius. An increase in *k* ^2^, which depends on domain scaling when diffusion coefficients are held constant, results in an increase in *n* and changes the predicted Turing pattern. Here, we derive a formula that predicts the Turing pattern observed on a prolate spheroidal surface which represents the SVZ.

### Prolate Spheroidal Surface

A prolate spheroid is created by rotating an ellipse about its major axis. It has a focal distance 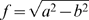, where *a* and *b* are the major and minor axes, respectively. Spheroidal coordinates are expressed as (*ξ*, *η*, *ϕ*) where *ξ* is the radial term; *η* = cos *θ*, where *θ* is the asymptotic angle with respect to the major axis; and *ϕ* is the rotation term.

To predict which pattern will emerge, the Helmholtz equation is expanded with respect to the prolate spheroidal coordinate system [19] resulting in
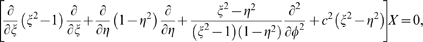
(4)where 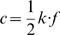. Because the Helmholtz equation (**∇** ^2^ **X**+*k* ^2^ **X** = 0) is separable in prolate spheroidal coordinates, we rewrite **X** in terms of **X** = *R*(*c*,ξ)*S*(*c*,η)**Φ**(**ϕ**), such that *S*(*c*,η), *R*(*c*,ξ), and Φ(ϕ) satisfy
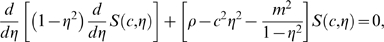
(5)

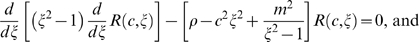
(6)

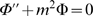
(7)where *m* and *ρ* are separation constants. Since multiple, discrete values of *ρ* are possible for a given *m* and *ρ* is also dependant on *c*, the notation will be *ρ_mn_*(*c*).

Because our domain is a prolate spheroidal surface, the radially-invariant solution is needed (i.e. 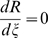). In order for Equation 6 to hold, 
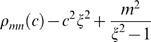
 must equal zero for a nontrivial solution. This results in
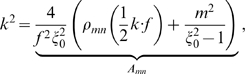
(8)where *ξ*_0_ is the spheroidal radius of the shell that conserves a surface area of 4π (comparable to the surface area of a unit sphere).

**Figure 2:**
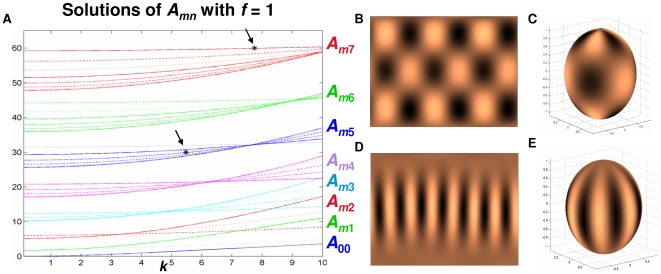
Computer modeling verification of spheroidal mode equation (Equation (8)). **A.** Graph of *A_mn_* for *n* = 0, ‥, 7 (different colors) and *m* = 0, ‥, *n* (different linestyles, beginning with *m* = 0 on the bottom and *m* = *n* on top) with *f* = 1. The black asterisks indicate *k* ^2^ = *A_mn_* = 30 (middle asterisk) and 60 (top right). The asterisk for *k* ^2^ = 30 corresponds to *A*_35_ predicting the pattern (3,5), whereas the asterisk for *k* ^2^ = 60 predicts the pattern (7,7). Discretizations of Equations 1*a*,*b* corresponding to *k* ^2^ = 30 and 60, as shown in Figures B–E agree with these predictions. **B.** Results of discretization of Equations 1*a*,*b* for δ = 0.013 (corresponding to *k* ^2^ = 30), α = 0.899, β = −0.91, γ = −0.899, *D* = 0.5319, *r*_1_ = 3.5, and *r*_2_ = 0. **C.** Projection of B onto a prolate spheroid (*f* = 1) such that top (bottom) edge of B maps to the north (south) pole of the spheroid. **D.** and **E.** Same as B and C except δ = 0.0065 (corresponding to *k* ^2^ = 60).

The significance of the formula in Equation 8 is that it relates a given domain size (controlled by *k* ^2^) and domain shape (the eccentricity of the prolate spheroid controlled by *f*) to the arising pattern. To demonstrate this formula's ability to predict pattern formation, the system (Equations 2a, b) is discretized similar to that of a sphere [18]. A forward-Euler finite difference scheme is used and *u* and *v* are discretized such that *u*(η, ϕ) = (−1+*h*_1_dη, *h*_2_dϕ) where *h*_1_ = 0,‥,34, and *h*_2_ = 0,‥,68. The continuity with respect to η around the north and south poles is maintained as described in [18] and periodic boundary conditions are used for ϕ. In Figure 2A, *A_mn_* is plotted for *n* = 0,‥,7 and *m* = 0,‥, *n*. Numerous simulations were executed and two are shown here. The first simulation (Figures 2B and 2C) corresponds to *k* ^2^ = 30. When *k* ^2^ = 30 (asterisk in middle) is plotted on the *A_mn_* vs. *k* graph (Figure 2A), *k* ^2^ corresponds with *A*_35_ and predicts a (3, 5) pattern that agrees with the numerical simulation (Figures 2B and 2C). The second simulation corresponds to *k* ^2^ = 60 (Figure 2D and 2E) and when plotted (top right asterisk) on the *A_mn_* vs. *k* graph, predicts a (7, 7) pattern that is observed in the numerical simulation.

## Results

A few key observations will be addressed. Qualitatively, as the domain scaling (i.e., *k* ^2^) increases, the resulting pattern becomes more elaborate. This result correlates with observations that relate the surface area of the VZ (or size of the founding radial glial cell population) to cortical surface area and hence, to the elaboration of the cortical pattern. The elaboration spans from no pattern observed for smaller surface areas of the VZ (and smaller-scaled cortices) to elaborate patterns observed for larger surface areas of the VZ (and larger cortices) [20].

### Sectorial and Transverse Sulci

**Figure 3:**
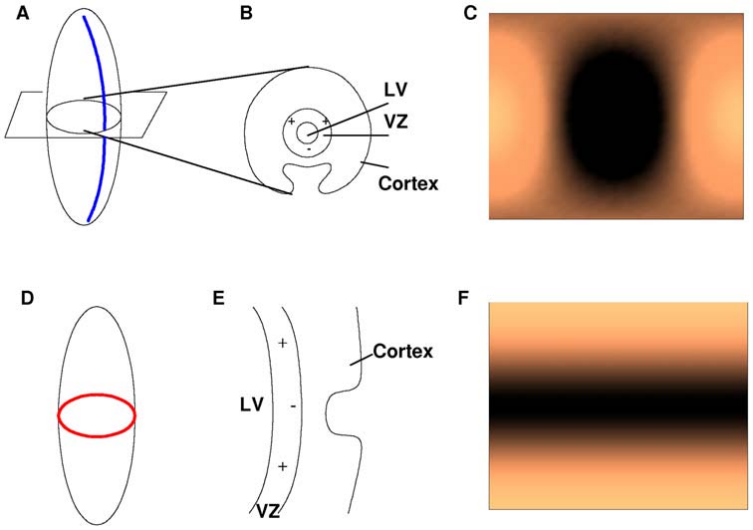
Determination of prolate spheroidal harmonic needed to form a sectorial and a transverse sulcus. **A.** A prolate spheroid containing one sectorial sulcus. **B.** A crossection of Figure A displaying the necessary pattern for a sectorial sulcus to develop. Plus signs indicate areas of activation of the creation of IP cells, whereas negative signs denote nonactivation. **C.** Spheroidal harmonic (1,1) which matches the pattern needed for a sectorial sulcus to develop. The top (bottom) edge maps to the north (south) pole and the left and right edges connect. **D.** A prolate spheroid containing one transverse sulcus. **E.** A crossection of Figure D displaying the necessary pattern for a transverse sulcus to develop. Plus signs indicate areas of activation of the creation of IP cells, whereas negative signs denote nonactivation. F. Spheroidal harmonic (0,2) which matches the pattern needed for a transverse sulcus to develop.

The model presented here addresses the directionality of the initial sulci formed. In order to use the predictive power of the proposed prolate spheroidal harmonic system, sulci need to be formulated in terms of prolate spheroidal harmonics. Since the initial sulcal formations mimic stripes, only the prolate spheroidal harmonics resulting in striped patterns were studied. In order to form a sulcus, the gyral banks on either side of the sulcus need to be created. In terms of the production of IP cells, the areas on either side of the sulcus will need to be ‘activated’ while the area of the sulcus is ‘not activated’ (see Figure 3B and 3E).

The two sulcal directions considered are sectorial and transverse. Sectorial sulci extend in the direction from the frontal lobe around the Sylvian fissure to the temporal lobe. This represents the direction of the major axis of the prolate spheroid approximating the lateral ventricle as shown in Figure 3A. The alignment of sulcal pits (deepest part of sulcus) along the major axis of the lateral ventricle in the human has been shown [21]. In terms of spheroidal harmonics, the pattern of IP cells needed to create sectorial sulci is (*m*, *n*) = (1,1) for 1 sulcus (Figure 3C), (2,2) for 2 sulci, and so forth. Transverse sulci form in the direction of rings around the VZ as shown in Figure 3D. This direction corresponds to (0,2) for 1 sulcus (Figure 3F), (0,4) for 2 sulci, and so forth. In each species displaying a cortical pattern, a number of sectorial sulci (or sulcal pits) are observed. The exact number of sectorial sulci is not the focus here. Of interest, rather, are the occurrence of a transverse sulcus, the transition from transverse to sectorial sulci, and the role of lateral ventricular eccentricity.

**Figure 4:**
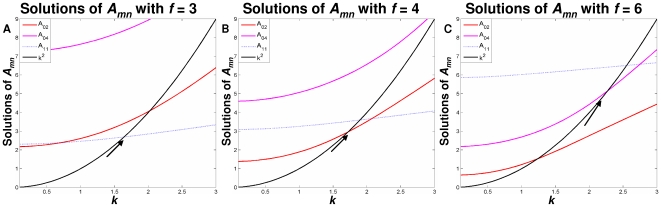
Bifurcations occurring between sectorial and transverse curves. **A.** An illustration of the scenario of order *A*_11_, *A*_02_, *A*_04_, which occurs for fixed focal distances up to 3.7. **B.** Scenario of order *A*_02_, *A*_11_, *A*_04_, which occurs for focal distances 3.7 to 5.6. **C.** Scenario of order *A*_02_, *A*_04_, *A*_11_, which occurs for focal distances 5.6 to 6.9.

For *f* = 3 (Figure 4A), as the domain scaling (*k* ^2^) increases, *A*_11_ is reached first, followed sequentially by *A*_02_ and *A*_04_. This sequence corresponds to a sectorial sulcus forming first. If the focal distance is increased, e.g. if *f* = 4 (Figure 4B), there is a shift in the *A_mn_* curves and, as *k* ^2^ increases, *A*_02_ will now occur before *A*_11_. This results in a transverse sulcus forming before the first sectorial sulcus. A further increase in focal distance to *f* = 6 (Figure 4C) again shifts the *A_mn_* curves, so that *A*_04_ now occurs before *A*_11_. Two transverse sulci will now form before a sectorial sulcus is created. These scenarios illustrate how focal distance plays a role in determining the order of pattern formation.

## Discussion

**Figure 5:**
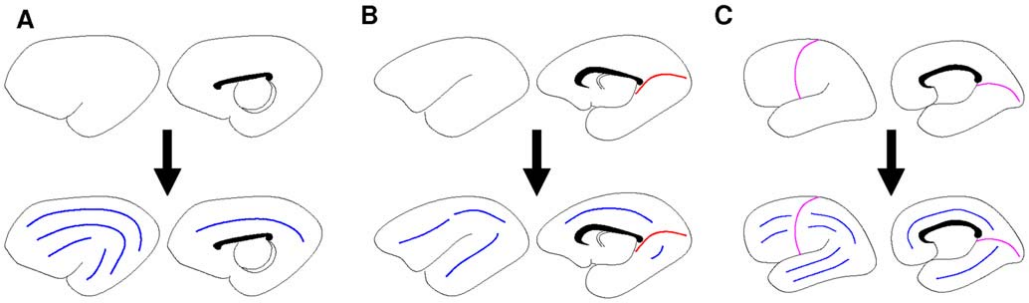
Predicted development of folds in cortices of 3 species. The sulcal patterns produced based on the lateral ventricular geometry shown in Figure 1, and the resulting bifurcations shown in Figure 4. The top row shows the formation of transverse sulci (red curve in B corresponding to the pattern *A*_02_, magenta curves in C corresponding to *A*_04_). The bottom row shows the subsequent creation of a certain number of sectorial sulci (blue curves) for **A:** cat, **B:** lemur, **C:** human.

The GIP model illustrates how sulcal placement and directionality is related to changes in focal distance. In order to determine the effect of changes in focal distance on cortical pattern formation, the evolutionary development of cortical patterns was examined. The lateral ventricle is a *c*-shaped cavity with an anterior horn that extends into the frontal lobe of the hemisphere and an inferior pole that enters the temporal lobe [11]. During the critical stages of brain development the volume of the lateral ventricle increases [22] which also increases the surface area of the lateral ventricle (i.e. k^2^ increases) . Also, as species have evolved the neocortex has expanded, resulting in major evolutionary advances [23]. As the frontal and temporal lobes expand, the lateral ventricle extends into the lobes increasing the lateral ventricular eccentricity resulting in changes in the cortical pattern obtained. For example, overlaying an evolutionary ladder on the scenarios described in Figure 3 implies that the cortices of species on the lower rungs of the evolutionary ladder, such as the cat, do not display transverse sulci before the formation of sectorial sulci (Figures 4A and 5A). Following this evolutionary ladder, at some point the first transverse sulcus appears, as shown in Figure 4B. This second stage is representative of the formation of the calcarine sulcus in species such as the lemur (Figure 5B). Further along the evolutionary ladder, the second transverse sulcus appears (Figure 4C). This is representative of the central sulcus found in higher order primates such as the human (Figure 5C).

For humans, this predicted ordering of sulcal formation correlates well with what has been observed during development through the examination of naturally aborted fetuses [24] and MRI study on preterm infants [25]. The first sulci to appear are the anterior calcarine and central sulcus which are in the transverse direction (blue lines in Figure 5C). This is followed by the formation of the superior and inferior frontal sulci, superior and inferior temporal sulci, the intraparietal sulcus and the cingulated sulcus; all which form in the sectorial direction (red lines in Figure 5C).

The GIP model also provides a plausible explanation for the development of the central sulcus. Lemurs and humans are both members of the primate order. The lemur is of the suborder prosimian, which is the most primitive of the primates [26]. Most prosimians can be distinguished from anthropoids, the higher primates, by the absence of the central sulcus [26]. Therefore, this model links evolutionary development, through the lateral ventricular eccentricity, to the development of the central sulcus.

The GIP model is a theoretical model that builds upon the ideas of the IP model. One argument that has been presented against the intermediate progenitor model is that an “elaborately choreographed set of developmental instructions [regulating the production of IP cells] would be required to account for the tremendous complexity of human cortical convolutions” [7]. The beauty of the GIP model is that it provides an uncomplicated approach that relates to a biologically plausible mechanism of pattern formation. It uses chemical morphogens that may be governed by specific genes to control IP cell production, resulting in the ability to predict the placement and directionality of sulcal pattern formation.

The GIP model reveals the role that the global shape of the lateral ventricle has on the positioning of the initial sulci during cortical development. This model explains the development of the initial folds, particularly how two transerve sulci can form before any sectorial sulci in the human. There are many sulci, such as the precentral and postcentral sulcus, that form after this event which are not in the scope of this present work. Also, we believe the Sylvian fissure is formed by the *c*-shape of the lateral ventricle, which is not applicable to the model.

Lateral ventricular shape, or shape of any nontrivial object, is not easy to quantify. The GIP model approximates the lateral ventricle with a prolate spheroid allowing the capture of key shape characteristics in *one* parameter, the focal distance (*f*). This approximation also gives the resulting patterns in terms of prolate spheroidal harmonics which contain an order based on the prolate spheroidal indices, *m* and *n*. The Helmholtz equation could also be solved on a given triangulated mesh representing the lateral ventricle resulting in a set of eigenvalues and eigenfunctions. The eigenfunction whose associated eigenvalue produces diffusion-driven instability would be the predicted pattern formed. A drawback of this latter approach occurs when comparing predicted patterns from different triangulated meshes. Since each mesh has its own parameterization there is no way of knowing which shape characteristic is responsible for the change in pattern formation.

Although changes in the volume of the lateral ventricle in humans during the developmental stages are documented [22], quantified data on the size and shape of the lateral ventricle during these critical stages is lacking. Further investigations into the size and shape of the lateral ventricle during developmental stages across species are needed. Such parameters could then be incorporated into the GIP model to test its cortical patterning predictions for specific species.

Also, further investigations into how the production of IP cells is regulated (i.e. how the activator and inhibitor interact) would enhance this model. Several genes (*Pax6*, *Ngn2*, and *Id4*) have been shown to modulate the production of IP cells in mice [10]. Further studies into how this modulation occurs, and if this modulation changes evolutionarily, could be incorporated into the reaction kinetics in the GIP model enhancing the cortical patterning predictions.

In conclusion, this chemically-based mathematical model (the global intermediate progenitor (GIP) model) extends the intermediate progenitor model [5], which describes local phenomena, to encapsulate *global* characteristics. In doing so, the GIP model shows how the global shape of the lateral ventricle, which drives the shape of the VZ, plays a key role in cortical pattern development. This model is able to capture changes in VZ shape along with the complementary role of domain scaling in only two parameters: 1) the focal distance of the prolate spheroid approximating the lateral ventricle, and 2) *k* ^2^, which is dependent on domain scaling, as given by the formula in Equation 8. The model also has the ability to predict why the cortex of certain species may have little or no folding, and it accounts for the order and directionality of the sulci formed in different species. We consider this model a first step toward a chemically driven and mathematically predictive explanation of cortical folding development across species.
